# The p97-FAF1 Protein Complex Reveals a Common Mode of p97 Adaptor Binding*

**DOI:** 10.1074/jbc.M114.559591

**Published:** 2014-03-11

**Authors:** Caroline A. Ewens, Silvia Panico, Patrik Kloppsteck, Ciaran McKeown, Ima-Obong Ebong, Carol Robinson, Xiaodong Zhang, Paul S. Freemont

**Affiliations:** From the ‡ Centre for Structural Biology, Department of Life Sciences, Imperial College London, South Kensington Campus, London SW7 2AZ, United Kingdom and; § Department of Chemistry, University of Oxford, South Parks Road, Oxford OX1 3TA, United Kingdom

**Keywords:** ATPases, Electron Microscopy (EM), Fas, Isothermal Titration Calorimetry, Protein Cross-linking, FAF1, p97

## Abstract

p97, also known as valosin-containing protein, is a versatile participant in the ubiquitin-proteasome system. p97 interacts with a large network of adaptor proteins to process ubiquitylated substrates in different cellular pathways, including endoplasmic reticulum-associated degradation and transcription factor activation. p97 and its adaptor Fas-associated factor-1 (FAF1) both have roles in the ubiquitin-proteasome system during NF-κB activation, although the mechanisms are unknown. FAF1 itself also has emerging roles in other cell-cycle pathways and displays altered expression levels in various cancer cell lines. We have performed a detailed study the p97-FAF1 interaction. We show that FAF1 binds p97 stably and in a stoichiometry of 3 to 6. Cryo-EM analysis of p97-FAF1 yielded a 17 Å reconstruction of the complex with FAF1 above the p97 ring. Characteristics of p97-FAF1 uncovered in this study reveal common features in the interactions of p97, providing mechanistic insight into how p97 mediates diverse functionalities.

## Teaser

**Background:** p97 function is facilitated by a huge array of varied adaptor proteins, including FAF1.

**Results:** FAF1 binds over the AAA rings of p97 alone and in a trimer-hexamer arrangement.

**Conclusion:** p97 and FAF1 bind with a similar affinity and in a similar conformation to other p97-adaptor complexes.

**Significance:** p97 binds multiple different adaptors by a common mode.

## Introduction

p97 (also known as VCP and Cdc48) is a ubiquitous member of the AAA^2^ ATPase protein family essential for cell viability (1). The active form of p97 is a homohexamer in which each monomer consists of three domains, two AAA domains, D1 and D2, arranged as two concentric rings, and an N-terminal domain that lies in the plane of the D1 ring in almost all crystal structures (2, 3). p97 is involved in a large array of processes throughout the cell cycle (4), which include endoplasmic reticulum-associated degradation (ERAD) (5), autophagy (6), mitochondrial-protein degradation (7), membrane fusion (8), and DNA damage repair (9).

The diverse functions of p97 are linked to its role in the ubiquitin-proteasome system, a process by which polyubiquitylated proteins are delivered to the proteasome for degradation. A diverse range of adaptor proteins with ubiquitin-binding sites directly interact with p97 and facilitate the recognition of ubiquitylated target proteins, which results in their degradation by the ubiquitin-proteasome system or in their recycling as part of distinct regulatory mechanisms (1, 4). The p97 adaptor proteins provide scaffolds with additional interaction motifs, regulating the assembly of p97-multiprotein complexes, as well as correctly orienting substrates for further processing (10). One particularly well studied adaptor is Ufd1-Npl4 (also known as UN), which is required for p97 function in ERAD (1), but the largest group of p97 adaptor proteins are the ubiquitin-regulatory X (UBX) domain-containing proteins, which include p47 and Fas-associated factor 1 (FAF1) (11).

FAF1 is best known as a component of the Fas-apoptosis pathway as it binds the death domain of Fas and promotes apoptosis in mammalian cells (12). In addition to a UBX domain, FAF1 contains a predicted coiled-coil domain (10), a ubiquitin-associating (UBA) domain and a number of other domains that link FAF1 with the ubiquitin-proteasome system. Together with p97, FAF1 is involved in mediating the degradation of ERAD substrates through binding of Lys-48- and Lys-63-linked polyubiquitylated substrates via its UBA domain (13, 14). FAF1 has also been found to interact with the major transcription factor NF-κB (p65 subunit), preventing it from entering the nucleus to activate gene transcription (15). The NF-κB heterodimer is normally retained in the cytoplasm by the regulator IκBα until phosphorylation and ubiquitylation of IκBα, which leads to its degradation via the ubiquitin-proteasome system (16). FAF1 (13) and p97 (17) have both been implicated in mediating IκBα degradation, via unknown mechanisms, indicating the importance for the p97-FAF1 complex. These roles of p97-FAF1 complex and FAF1 itself make them potential therapeutic targets for the treatment of cancer and neurodegenerative disorders (18).

Currently, there is no structural or biophysical information about the full-length p97-FAF1 complex, although crystal structures of the UBX domain of FAF1 (FAF1-UBX) bound to the N-terminal domain of p97 have been determined (19, 20). Furthermore, there are inconsistent data over whether UN is required for FAF1 binding to p97 as part of a hierarchical assembly mechanism of p97 adaptor complexes (13, 19–22). In this study, we have carried out a detailed biochemical, biophysical, and electron microscopy characterization of the p97-FAF1 complex. Our study unambiguously shows that a full-length p97-FAF1 complex can be stably formed in the absence of UN and we have obtained a cryo-EM model of p97-FAF1 at ∼17 Å. Together, our data allow us to propose a common mode of interaction for p97 adaptors despite differences in domain architecture, suggesting a conserved mechanism of action for p97-adaptor complexes.

## MATERIALS AND METHODS

### 

#### 

##### Expression and Purification

Full-length and truncated constructs of murine p97 and adaptor proteins were prepared as follows: C-terminally His-tagged full-length p97 (residues 1–806) in PET22b, N-terminally His-tagged full-length FAF1, and FAF1-ΔUBX(1–560) in pProEx, GST-tagged FAF1-CC-UBX(491–649), FAF1-CC(491–560), and FAF1-UBX(550–649) in pGEx, and untagged full-length p97, C-terminally His-tagged full-length FAF1, untagged Ufd1 and His-tagged Npl4 in PET28b. C-terminally His-tagged FAF1 was used for nanogold labeling, whereas N-terminally His-tagged FAF1 was used for all other experiments. All proteins were produced recombinantly in Rosetta 2 cells. The cultures were grown to an *A*_600_ of 0.4–0.8 and induced with 1 mm isopropyl 1-thio-β-d-galactopyranoside overnight at 22 °C, except for the UBX constructs, which were expressed by autoinduction. His-tagged proteins were purified by resuspending the bacterial pellet in His buffer A (50 mm HEPES, 500 mm KCl, 20 mm imidazole, pH 7.4), lysing the cells by sonication and then loading the cleared lysate onto a HiTrap nickel affinity column (GE Healthcare). For pulldown experiments, cells expressing untagged p97 were lysed and prepared in the same way and then loaded onto the column after the first protein was loaded and washed. The protein was then eluted using an imidazole gradient (to 500 mm imidazole over 6 to 8 column volumes). Full-length FAF1 was further purified by ion exchange on a HiTrap Q HP column (GE Healthcare) (20 mm phosphate, pH 7.4, 50–1000 mm KCl). Cells expressing Ufd1 and Npl4 were co-lysed and purified together. For GST-tagged proteins, cell pellets were resuspended in GST buffer A (20 mm Tris, 500 mm KCl, 5 mm 2-mercaptoethanol, pH 8), lysed as described previously, and the cleared lysate was loaded onto a GSTrap column (GE Healthcare). The protein was then eluted with glutathione (20 mm). The GST tag was cleaved using GST-tagged HRV 3C during dialysis into GST buffer A overnight, the GST-affinity purification was then repeated to isolate the cleaved protein. After the affinity chromatography, and ion exchange in the case of full-length FAF1, all proteins were further purified by size-exclusion chromatography. All size-exclusion chromatography was done using SEC buffer (20 mm HEPES, 150 mm KCl, pH 7.4) and using columns appropriate for the size of the proteins, either Superose 6, Superdex 200, or a Superdex 75. An analytical Superose 6 (GE Healthcare) was used for all p97-FAF1 complex samples. For the p97/FAF1/UN binding studies, the components were separately purified, mixed in the appropriate concentrations, in a ratio of 1:1:0.5, and run on an analytical Superose6 after 15 min of incubation on ice.

##### Microscale Thermophoresis

Microscale thermophoresis (MST) was carried out on a Monolith NT.115 instrument (NanoTemper Technologies) (23). Full-length FAF1 and FAF1-ΔUBX were labeled with NanoTemper495-*N*-hydroxysuccinimide blue/red dye per the manufacturer's instructions. 16 sample points were used in standard treated capillaries and containing 0.06 μm labeled FAF1 and full-length p97 between 135 and 0.0041 μm in SEC buffer (20 mm HEPES, 150 mm KCl, pH 7.4). The experiment was carried out at 30 °C using 80% LED power (470 nm) and 80% laser power. The fraction of FAF1 bound to p97 was calculated by subtracting the unbound hot/cold value and dividing by the difference between the bound and unbound hot/cold value of the full-length FAF1 experiment. An average of two separate experiments was taken, and the *K_d_* was estimated for full-length FAF1 by fitting the data with the Hill equation in Origin 8.6.

##### Sedimentation Velocity Analytical Ultra-centrifugation

All protein samples were gel filtrated into AUC buffer (25 mm HEPES, 200 mm KCL, 0.5 mm tris(2-carboxyethyl)phosphine, pH 7.4) prior to analysis. Sedimentation velocity ultra-centrifugation (SV-AUC) was carried out using a Beckman Optima XL-I analytical ultracentrifuge at 20 °C using an AnTi50 rotor. Interference optics data sets were collected at rotor speeds of 30,000 rev/min in sector cells with column heights of 12 mm. Scans were recorded at 1-min intervals over 15 h. The observed sedimentation boundaries were fitted to yield a *c*(s) plot according to the Lamm equation using SEDFIT (version 14.1) (24, 25). The best-fit frictional ratio was calculated using SEDFIT by applying a fixed resolution of 200 and floating the frictional ratio, the meniscus and the base line until the overall root mean square deviations and visual appearance of the fits between observed and calculated sedimentation boundaries were satisfactory. Buffer density was measured using an Anton Paar DMA 5000 Density meter and was found to be 1.009946 g/ml.

##### Isothermal Titration Calorimetry

For the isothermal titration calorimetry (ITC) experiments, the cell protein (full-length p97), at 10–50 μm, and the titrant protein (full-length FAF1 or FAF1 truncations), at 7.5 to 10 times the cell protein concentration, were dialyzed into ITC buffer overnight (20 mm HEPES, 150 mm KCl, pH 7.4). The ITC experiment was performed on a VP-ITC instrument (MicroCal, GE Healthcare), with 30 × 10 μl injections at 300-s intervals and at 30 °C. The results were analyzed using Origin (version 7). A theoretical curve was fitted to the data using least-squares fitting to estimate stoichiometry, *K_a_* (from which the reciprocal, *K_d_*, was calculated), and Δ*H* and an average of two measurements was taken.

##### Mass Spectrometry

The full-length FAF1 sample at 10 μm was dialyzed into 200 mm ammonium acetate (pH 7.5), and the native mass spectrometry was carried out on a Q-ToF mass spectrometer modified for high mass detection.

##### Chemical Cross-linking

For chemical cross-linking experiments, 40 μl of 15 μm full-length FAF1 and various truncations in SEC buffer were incubated at room temperature with 0.2 μl of 50 μm ethyl glycol bissuccinimidyl succinate in dimethyl sulfoxide. The reaction was stopped after 15 min by the addition of 1 μl of 1 m Tris and left for 5 min, and the samples were then analyzed by SDS-PAGE electrophoresis.

##### Electron Microscopy

To prepare the cryo-EM grids, 5 μl of purified p97-FAF1 at 0.2 mg/ml were applied to glow-discharged holey-carbon grids, which were then blotted and flash frozen in liquid ethane using a Vitrobol (FEI). Data were collected on a CM200 (Philips) electron microscope, operating at 200 kV, under low dose conditions, at 50,000× magnification, and at 2–4 μm nominal defocus. For the nanogold labeling, a 0.05 mg/ml complex of C-terminally His-tagged FAF1 and untagged p97 was mixed with 5 nm of nickel-nitrilotriacetic acid nanogold. The sample was applied to glow-discharged continuous-carbon grids and stained with 2% uranyl acetate solution. Images were collected using a Teitz camera at the Imperial College Electron Microscopy Centre.

Data processing was carried out using Imagic (version 5) (26), unless stated otherwise. The micrographs were coarsened by a factor of 2, leaving a pixel size of 3.52 Å/pixel, and contrast transfer function-corrected. 60,032 particles were picked interactively using BOXER in EMAN, and band-pass filtered between 10 and 200 Å and then normalized and masked. For the p97-FAF1 data set, reference-free class averages were created from successive rounds of multivariate statistical analysis and alignment (brute force alignment (31). For the four-dimensional data processing, multiple side view class averages were used to make initial three-dimensional models with C6 symmetry. These were refined by successive rounds of competitive alignment and projection matching to separate the data set. Models representative of p97-FAF1 complex were judged visually, and the particles that aligned to them were extracted and processed separately. A single side view was selected and used to make an initial 6-fold model that was refined without symmetry by progressive rounds of alignment and projection matching. The process was then repeated with 3-fold symmetry imposed throughout the processing. The final C3 p97-FAF1 protein complex model has been deposited on the EMDB website (accession code EMD-2319).

##### Model Fitting

Atomic structures were fit into the cryo-EM density maps using Chimera and VEDA (visual environment for docking algorithm).

## RESULTS

### 

#### 

##### Full-length FAF1 Binds p97 and Requires FAF1-UBX Domain

**FIGURE 1.**
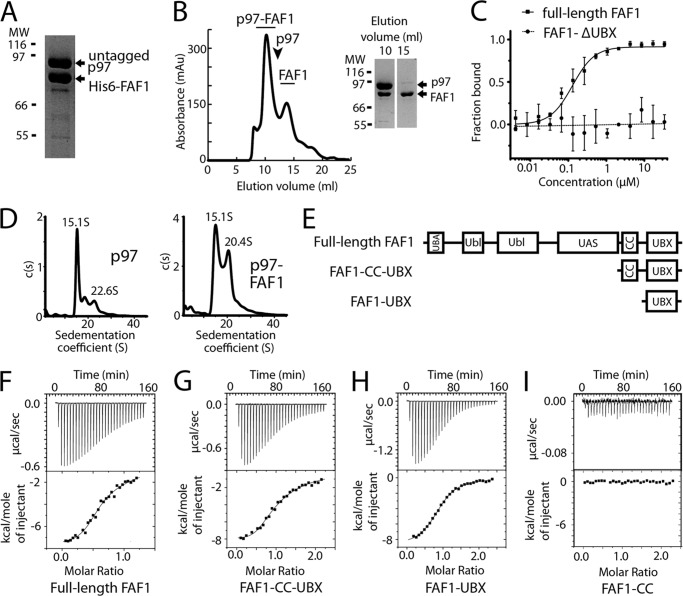
**p97 binds FAF1 and forms a stable complex.** *A*, SDS-PAGE of a nickel-affinity chromatography experiment using His-tagged FAF1 and untagged p97 shows both components in the elution peak. *B*, the elution profile and SDS-PAGE of the gel filtration of p97-FAF1 complex on an analytical Superose 6 (GE Healthcare) shows that they form a stable complex, the elution volume of p97 alone is indicated. *C*, MST of full-length FAF1 (*squares* and *solid line*) and p97 shows they bind with a *K_d_* of 0.13 μm (± 0.02 μm). The MST of FAF1-ΔUBX and full-length p97 shows no binding (*circles* and *dotted line*). The average of two repeats is shown in both cases, with the best fit curve and the concentration of titrant (μm) plotted against fractions bound. *D*, the *c*(*s*) distribution analysis of SV-AUC results for p97 at 2 mg/ml and FAF1/p97 complex at 2 mg/ml. *Arrows* indicate S value for each discrete peak. *E*, a schematic of the domain organization of full-length FAF1, FAF1-CC-UBX, and FAF1-UBX. Isothermal titration calorimetry was carried out against full-length p97 using full-length FAF1 (*F*), FAF1-CC-UBX (*G*), FAF1-UBX (*H*), and FAF1-CC (*I*). The average best fit values of two repeats gave values as follows: full-length FAF1, *K_d_* values 0.8 μm ± 0.0 μm and 19 μm ± 9 μm, stoichiometry of 1 to 0.5 ± 0.1 and 0.5 ± 0.2; FAF1-CC-UBX, *K_d_* values 0.7 μm ± 0.2 μm and 12 μm ± 3 μm, stoichiometry of 1 to 0.6 ± 0.1 and 0.6 ± 0.2; FAF1-UBX, *K_d_* 3.9 μm ± 0.4 μm, stoichiometry of 1 to 0.98 ± 0.1. No binding was observed for FAF1-CC.

We first investigated the binding of recombinant full-length FAF1 to p97. Nickel-affinity chromatography showed that His-tagged FAF1 was able to pull down untagged p97 (Fig. 1*A*), indicating an interaction. Size-exclusion chromatography of the p97-FAF1 complex showed a symmetric elution peak significantly shifted from that of p97 alone confirming the formation of a stable complex (Fig. 1*B*). Next, the binding of the full-length proteins was analyzed quantitatively using MST, which revealed a *K_d_* of 0.13 μm (± 0.02 μm) (Fig. 1*C*). A control experiment using FAF1 without its UBX domain (FAF1-ΔUBX) and full-length p97 displayed a flat line indicating no measurable binding and confirming that the binding detected with full-length FAF1 was specific. To elucidate further details on the assembly of the p97-FAF1 complex, SV-AUC was performed on p97 and p97-FAF1 (Fig. 1*D*). Full-length p97 displayed one main species with a sedimentation coefficient of 15.1 S, which, using a best-fit frictional ratio of 1.48, corresponded to a molecular weight of ∼532 kDa in accordance with p97 hexamer size. A smaller peak at 22.6 S was also observed corresponding to the molecular mass of a p97 dodecamer, ∼991 kDa. In contrast, analysis of SV-AUC data for p97-FAF1 showed the presence of two main species. The first species matched the sedimentation value of the p97 hexamer of 15.1 S, whereas the second species, which represented the p97-FAF1 complex, was characterized by a sedimentation value of 20.4 S. Assuming a frictional ratio similar to that of p97, this sedimentation value corresponds to a molecular mass of ∼789 kDa and indicates that FAF1 interacts with p97 with a stoichiometry of 3:6. We next carried out a quantitative binding analysis by ITC using full-length p97 and either full-length FAF1 or FAF1 truncations (Fig. 1*E*). The full-length FAF1 binding data preferentially fitted a two-site binding model (Fig. 1*F*), with *K_d_* values of 0.8 μm (± 0.0 μm) and 19 μm (± 9 μm) with a stoichiometry of 1:0.5 (p97/FAF1) for both sites. The *K_d_* of the tighter binding site is consistent with the *K_d_* calculated by MST. This stoichiometry is indicative of a hexamer-trimer p97-FAF1 arrangement. Given the increased uncertainty in fitting more parameters, we could not rule out a one-site model, with an estimated *K_d_* of 5.2 μm (± 0.1 μm) and stoichiometry of 1 to 0.8 (± 0.03).

Next, a FAF1 construct comprising the coiled-coil and UBX domains (FAF1-CC-UBX) was used in binding studies to p97. The FAF1-CC-UBX construct exhibited similar thermodynamic characteristics as full-length FAF1 (Fig. 1*G*). A two-site binding model could be fitted with *K_d_* values of 0.7 μm (± 0.2 μm) and 12 μm (± 3 μm) and stoichiometries of 1:0.6. Fitting of a one-site model with a *K_d_* of 4.9 μm (± 1.8 μm) and a stoichiometry of 1 to 0.89 ± 0.3 was also possible. Conversely, FAF1-UBX binding to p97 exhibited a clear one-site binding model with a *K_d_* of 3.9 μm (± 0.4 μm) and a stoichiometry of 1:0.98 (Fig. 1*H*). The coiled-coil of FAF1 does not directly contribute to p97 binding as no binding was detected for a construct containing the coiled-coil domain alone (FAF1-CC) (Fig. 1*I*). These data clearly show that FAF1, via its UBX domain, is able to bind p97 to form a stable complex in the absence of other interaction partners.

##### FAF1 Has the Capacity to Form Higher-order Oligomers

**FIGURE 2.**
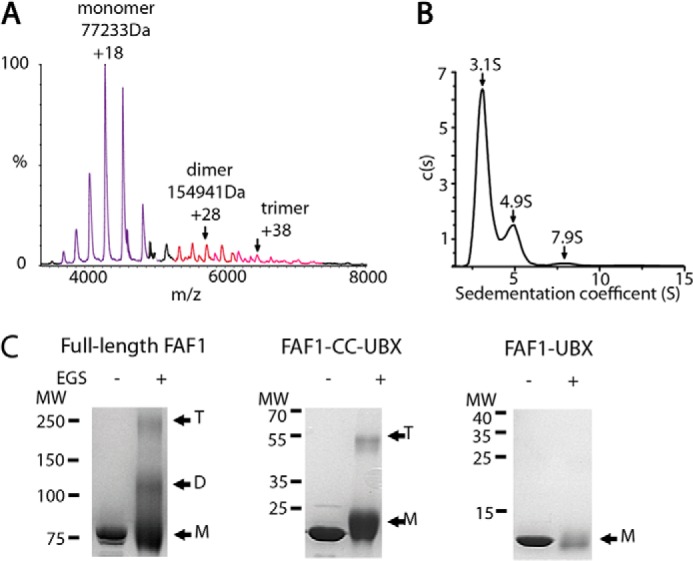
**FAF1 has the capacity to form oligomers via its coiled-coil region.** *A*, native mass spectrometry of full-length FAF1. The peaks corresponding to monomer (*blue*), dimer (*red*), and trimer (*pink*) species are labeled. *B*, the *c*(*s*) distribution analysis of SV-AUC results for FAF1 at 2 mg/ml. *Arrows* indicate S value for each discrete peak. *C*, cross-linking of full-length FAF1, FAF1-CC-UBX, and FAF1-UBX. Samples were incubated with or without ethyl glycol bissuccinimidyl succinate (*EGS*) for 15 min and then run on an SDS-PAGE gel. The molecular weight markers (*MW*), monomers (*M*), dimers (*D*), and trimer (*T*) bands are labeled.

To test whether FAF1 can form higher-order oligomers as suggested by our ITC data, we analyzed samples of purified FAF1 in solution by native mass spectrometry (Fig. 2*A*). The most abundant species in solution was monomeric FAF1, but dimers and trimers were also observed (Fig. 2*A*). Additionally, SV-AUC data confirmed that FAF1 was comprised of three species with sedimentation coefficients of 3.1 S, 4.9 S, and 7.9 S, which, using a best-fit frictional ratio of 1.98, correspond to molecular masses of a monomer, dimer, and trimer, ∼73, 144, and 280 kDa, respectively (Fig. 2*B*). To further probe the ability of FAF1 to form oligomers, we performed chemical cross-linking experiments on full-length FAF1 and a series of FAF1 truncations. Cross-linked products consistent with trimeric species were observed for full-length FAF1 and FAF1-CC-UBX but not FAF1-UBX (Fig. 2*C*). Taken together, these data show that full-length recombinant FAF1 has the capacity form weakly bound dimeric and trimeric species and that the coiled-coil region is necessary for oligomerization.

##### FAF1 Binds p97 and p97-UN in a Concentration-dependent Manner

**FIGURE 3.**
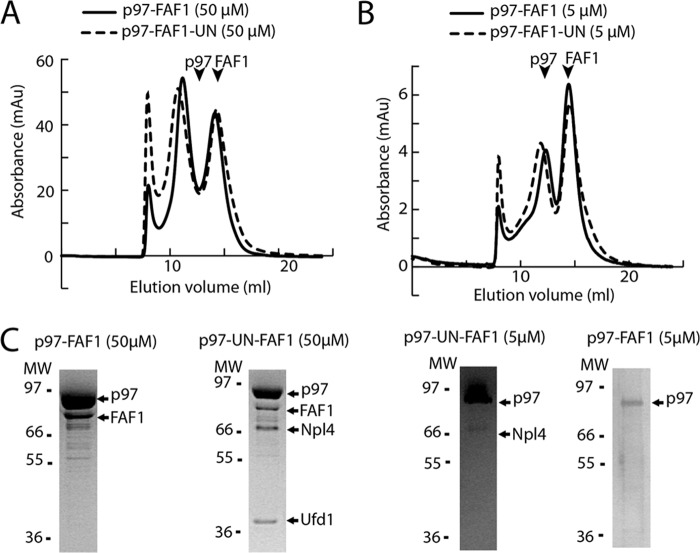
**p97 and FAF1 bind in a concentration-dependent manner and independently of UN.** *A*, analytical gel filtration elution profiles of mixtures of p97 and FAF1 without or with UN at 50 μm, elution volumes of the individual proteins are indicated. *B*, analytical gel filtration elution profiles of mixtures of p97 and FAF1 without or with UN at 5 μm, elution volumes of the individual proteins are indicated. *C*, SDS-PAGE gels of relevant samples of gel filtration fractions to analyze the contents of peaks. Molecular weight markers and the main bands are labeled. *mAu,* milliabsorbance units.

Our biochemical studies show that FAF1 can directly bind p97, whereas previous studies have suggested that the binding of the p97 adaptor UN is required for FAF1 binding (19, 22). To further investigate this, we carried out a series of size-exclusion chromatography experiments on p97 and FAF1 with and without UN, at various concentrations. We found that both FAF1 alone, and FAF1 and UN together could form stable complexes with p97 at high concentrations (50 μm) and that there was no prerequisite for UN binding (Fig. 3, *A* and *C*). At low concentrations (5 μm), FAF1 did not form a stable complex with p97, and FAF1 and UN together showed only small amounts of p97-UN complex (Fig. 3, *B* and *C*). These data show that FAF1 can bind p97 both independently and together with UN and that UN binding does not significantly enhance FAF1 binding to p97.

##### Cryo-EM Reconstruction of p97-FAF1 Complex

**FIGURE 4.**
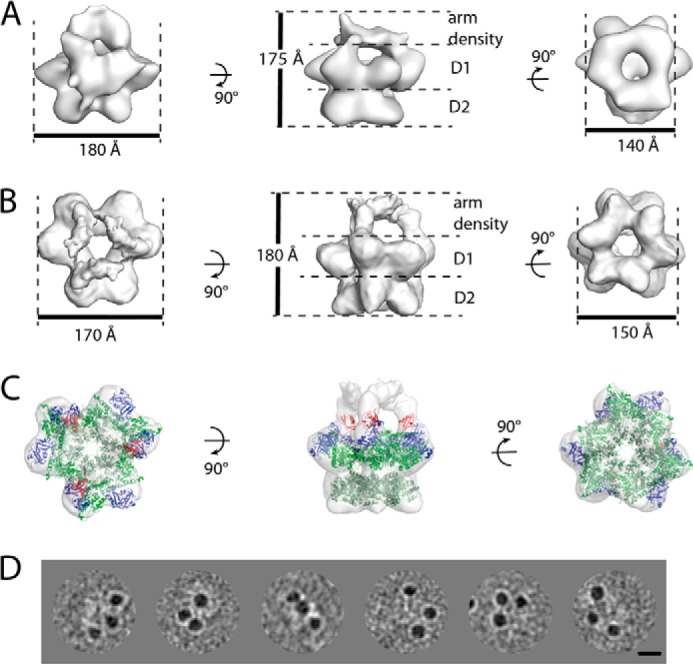
**Cryo-EM reconstructions of p97-FAF1.** *A*, the reconstruction obtained from p97-FAF1 particles after four-dimensional analysis with no symmetry applied during refinement. *B*, the p97-FAF1 reconstruction obtained with 3-fold symmetry imposed on the model throughout the refinement. Surface rendering of the *top*, *side*, and *bottom* view of the models, with respect to the symmetry axis, are displayed. The layers and dimensions of the model are labeled. *C*, atomic structures, based on Protein Data Bank codes 3CF2 and 3QQ8, were fitted into the cryo-EM reconstruction of p97-FAF1. Displayed is the surface rendering of the model and the FAF1 UBX domains (*red*) and the p97 N-terminal domain (*blue*), D1 (*green*), and D2 (*beige*) domains in their positions of best fit. *D*, C-terminal His-tagged full-length FAF1 in complex with full-length p97 was labeled with 5 nm nickel-nitrilotriacetic acid nanogold. Three gold particles around p97 rings could be seen.

To determine the quaternary arrangement of the p97-FAF1 complex, we used single-particle cryo-electron microscopy analysis. Nickel-affinity chromatography was used to form the p97-FAF1 complex by utilizing a His-tagged FAF1 to pull down untagged p97, followed by size-exclusion chromatography. The freshly purified protein (0.3 mg/ml) was used to make cryo-EM grids, which yielded ∼60,000 particles from 281 micrographs. To isolate different complex conformations within the EM data set, we used four-dimensional image processing and competitive alignment, which has previously been successful in obtaining cryo-EM reconstructions of p97-UN from conformationally heterogeneous samples (27). This involved creating 10 models with imposed 6-fold symmetry from reference free side view class averages. The models were concurrently refined using rounds of alignment and projection matching against the entire data set. After several rounds, it became clear that the data set contained a significant amount of unbound p97. The particles that aligned to the complex models were extracted from the data set resulting in ∼28,000 particles that were refined separately. Reference-free class averages were created, and a side view was selected and used to produce a 6-fold symmetric initial model. This initial model was refined without imposed symmetry over several rounds of alignment and projection matching until it stabilized (Fig. 4*A*). The final model consists of two rings, characteristic of p97, with a pseudo 3-fold symmetric density above the rings that make three connections with non-adjacent lobes of p97. A composition of the final model of six p97 and three FAF1 molecules is consistent with the biochemical analysis that suggests a p97:FAF1 stoichiometry of 6:3. As FAF1 has been shown to have only one p97 binding domain (13, 19), the observed density is unlikely to be a single FAF1 molecule. To verify the number of FAF1 molecules in the p97-FAF1 complex, we performed nanogold labeling of the complex. We observed three spots of high intensity associated with p97 hexamers confirming that three FAF1 protomers could bind to a p97 hexamer, although the 16-amino acid-long linker to the His tag on FAF1 is flexible, suggesting further localization of its binding site on p97 would be inaccurate (Fig. 4*D*). Although the possibility of adjacent p97-monomer FAF1 binding cannot be conclusively ruled out, our C1 model indicates FAF1 mostly binds to non-adjacent p97 subunits. Based on these observations, as well as the ITC and SV-AUC, the image processing was repeated with 3-fold symmetry imposed throughout. In the last three rounds of processing, a mask was imposed on the three-dimensional model that was based on the shape of the particle to further refine the model. The resultant model consists of two concentric six-lobed rings where every other lobe of the upper ring extends up into L-shaped arm densities above the rings (Fig. 4*B*). The model has an estimated resolution of ∼17 Å by the ½ bit criterion. Available crystal structures were fitted into the density to help assign the domains (Fig. 4*C*) with the double rings corresponding to the D1 and D2 rings of p97, respectively. The D2 ring is compact and displays distinct 6-fold symmetry similar to the p97 crystal structure, whereas the D1 ring is more open and displays clear 3-fold symmetry. The arm densities within the p97-FAF1 EM maps have a shape and size (32,000 Å^3^, ∼27 kDa) that cannot be explained with the presence of only the N-terminal domains of p97 (20-kDa each), suggesting that the arm densities contain some part of bound FAF1. By aligning the p97-FAF1 model with a copy rotated by ∼60°, the bound and unbound D1 domains can be compared. Extra density is visible above the bound lobes and in the plane of the unbound lobes. The unbound N-terminal domains can be fitted reliably into the extra density in the plane of the D1 ring. The N-terminal domains bound to FAF1 would pack on top of the D1 domains so that FAF1 UBX domain extends upwards into the arm density. The remaining density is therefore part of the rest of FAF1 with the N-terminal end of the UBX domain oriented to face it. These cryo-EM reconstructions reveal that FAF1 binds to p97 above the double rings of p97 and in agreement with the biochemical data, with a stoichiometry of 3 to 6.

## DISCUSSION

### 

#### 

##### FAF1 Binds p97 to Form a Stable Complex

FAF1 is an important member of the UBX domain-containing protein family (11). In association with p97, it mediates a number of important regulatory pathways, including NF-kB activation (13, 15, 18). We have performed a series of biochemical experiments to characterize the interaction of p97 with FAF1. Our pulldown assays, size-exclusion chromatography, MST, ITC, SV-AUC and cryo-EM experiments show that p97 can bind to FAF1 to form a stable complex independently of UN in agreement with a number of previous studies (13, 20, 21). The binding interface between the UBX domain of FAF1 and the N-terminal domain of p97 is highly similar to that of the UBX domain of p47, which also binds independently of UN (19, 20, 28). It has been proposed previously that binding of FAF1 to p97 requires binding of UN. Reasons for the discrepancy between our data and studies that could not detect binding of FAF1 and p97 in the absence of UN are unclear. The *K_d_* values we have obtained from several independent biophysical techniques for p97-FAF1 binding are comparable to those calculated in the presence of UN (0.8 μm compared with 2.3 μm for full-length FAF1; 3.9 μm compared with 8.4 μm for FAF1-UBX) (19, 22). The binding affinity of FAF1 for p97 is also similar to that of UN itself (1.7 μm for full-length UN), further suggesting that UN binds no more strongly to p97.

Our results show that, under specific conditions and concentrations of cofactors, p97, FAF1, and UN can form a ternary complex in agreement with other studies (19, 22, 29). Cryo-EM reconstructions of p97-FAF1 and p97-UN suggest that in both cases, the unbound N-terminal domains remain accessible for additional cofactors to bind, although not above the p97 rings. It has been observed using cryo-EM that p97-UN shows a high degree of flexibility with several conformations for UN possible, including coplanar to the D1 ring. It is conceivable that UN or FAF1 protomers may bind on the periphery of the p97 hexamer if they are sterically hindered from being above the p97 rings in the conformations they preferentially adopt when they bind p97 alone (27). This interference between the two adaptors when competing to bind on the p97 hexamer may explain the discrepancy in the stoichiometry calculated by ITC for p97 and FAF1 compared with p97-UN and FAF1 (1 to 0.5 compared with 1 to 0.18) (19). It has also been shown that FAF1 and UN do not bind each other directly to stabilize their interaction with p97 (19). Taken together, these data indicate that although UN and FAF1 can bind p97 at the same time they do not aid the binding of the other and could potentially hinder binding. Although knocking down Npl4 in cells reduced FAF1-induced ERAD model-substrate degradation (22), most key roles of p97-FAF1, similar to NF-κB activation, are not linked to UN function. For these reasons, it is likely that p97-FAF1 is the main functional complex in the p97-dependent cellular pathways of FAF1, although p97-FAF1-UN may still have a specific role, in which case, its quaternary arrangement will be an interesting question to investigate.

The concentration-dependent nature of the formation of p97-FAF1 complexes and comparable dissociation constants between p97-FAF1 and p97-UN leads to a speculative model for p97-cofactor assembly *in vivo*, where an increase in local concentration of cofactors bound at ubiquitylated target complexes would recruit p97 to specific target sites for the remodeling of those target substrates. Under these conditions, recruitment of other cofactors could also occur to form subsets of multiprotein complexes specific to a particular role but would be dependent on localized cofactor concentrations. This model would overcome the difficulty of preforming multiple p97 cofactor complexes given the large number of competing cofactors. Such a model would enable specific p97-cofactor complexes to be formed transiently where they are needed in contrast to preassembled complexes being targeted to sites of action, although it is possible that both scenarios operate.

##### Quaternary Arrangement of the p97-FAF1 Complex

Collectively, our data allow us to propose a model for the quaternary arrangement of p97-FAF1. Our cryo-EM model shows that FAF1 binds on top of the p97 hexamer similar to other p97 adaptors such as p47 and UN (27, 30). The majority of the mass of FAF1 is unaccounted for in our reconstruction. As FAF1 is a multidomain protein with regions of no predicted secondary structure, it is likely to be highly flexible. Any such density in our reconstructions would therefore be lost upon averaging. In our model (Fig. 4), the observed FAF1 density corresponds to the UBX domain and the preceding linker. The weak nature of the linker density and different appearance to the C1 model suggest that it is a flexible region, in agreement with secondary structure predictions. This may also explain why the oligomerizing coiled-coil region is also not visible and the FAF1 densities appear unconnected. The extended and flexible nature of FAF1 when bound to p97 could increase the accessibility of its additional binding sites, facilitating its proposed role as a scaffolding protein (14, 18).

We have established, using ITC, SV-AUC, and EM, that the p97-FAF1 complex has a 6:3 stoichiometry. Native mass spectrometry, chemical cross-linking, and SV-AUC reveal that FAF1 has the capacity to form weak dimers and trimers via its coiled-coil region in solution, accounting for the trimer-hexamer arrangement with p97. Our ITC data also suggest a two-site binding mechanism with the stronger binding event likely to be the FAF1 UBX domain binding to the p97 N-terminal domain, consistent with our MST results and data published previously (13, 19, 20). The predicted coiled-coil domain of FAF1 is required for the weaker second binding event, but it does not appear to interact directly with p97. One possibility is that the second binding event is caused by the oligomerization of FAF1 upon p97 binding, although further studies would be necessary to confirm this. Having three FAF1 protomers binding to a p97 hexamer increases the number of available polyubiquitin binding sites, allowing the binding of multiple ubiquitin chains or one chain at multiple sites. The Fas receptor, which also binds to FAF1, is known to trimerize upon Fas ligand binding, and it is interesting to speculate that a trimeric FAF1 bound to p97 reflects the oligomeric state of potential FAF1 target complexes. The 6:3 stoichiometry is the same as for p97-p47 adaptor complex despite little sequence identity other then conserved UBX and UBA domains (30). UN also binds p97 as a heterodimer to two separate N-terminal domains, and it has been speculated that other UBX proteins may oligomerize as they contain potential oligomerization domains (10). This common, multiple site-binding mode within p97 adaptor proteins may provide polyvalent substrate or cofactor binding and impose architectural constraints on the complex.

##### The p97-FAF1 Complex Reveals a Common Mode of p97 Adaptor Binding

**FIGURE 5.**
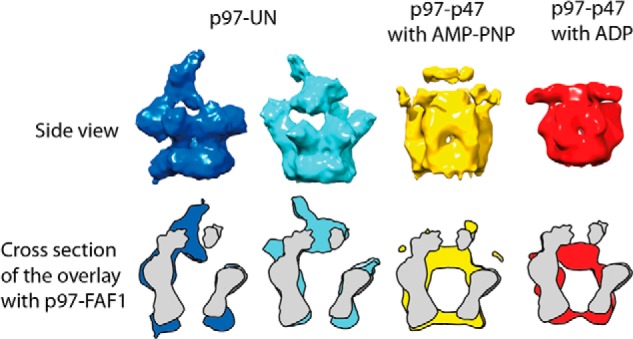
**Comparison of p97-FAF1 with p97-UN and p97-p47.** The p97-FAF1 model (*gray*) was compared with previously published models of two snapshots of p97-UN with no added nucleotide (*dark blue* and *light blue*) (27). The p97-FAF1 model was also compared with reconstructions of p97-p47 in the presence of AMP-PNP (*yellow*) and ADP (*red*) (30). The models are shown individually from the side with respect to the 6-fold symmetry axis, and the overlay is shown as a cross-section of the side view.

The p97 conformation in our p97-FAF1 reconstruction is comparable with that seen in the p97-UN complex with the diameter and shape of the D1 and D2 rings similar to our previously published p97-UN models with no added nucleotide (Fig. 5) (27). Interestingly, the positions of the extended densities from the p97 ring to the adaptor proteins are highly similar in both reconstructions. In both models, these densities correspond to N-terminal domains located above the plane of the D1 ring interacting with the UBX or UBD domains of FAF1 or UN, respectively. p97-FAF1, in comparison with previously published p97-p47 cryo-EM reconstructions in the AMP-PNP and ADP states (30), also shows the same overall p97 arrangement, with the N-terminal domains bound to the adaptor above the D1 ring. There are differences in the precise orientation of the N-terminal domain and AAA ring widths that are likely due to the different nucleotide states of the complex. Given the locked “in-plane” location of the N-terminal domain as observed in crystal structures (2, 3), it is interesting to speculate that a relocation of the N-terminal domain during the p97 ATP binding and hydrolysis cycle from above the plane of the ring to the side of the ring could provide the necessary force for target protein disassembly.

**FIGURE 6.**
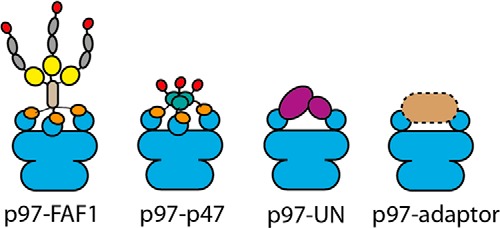
**A schematic for the common mode of p97 adaptor binding.** In the p97-FAF1 complex, three FAF1 protomers bind p97 (*blue*) via their UBX domains (*orange*) and oligomerize through coiled-coil domains (*brown*), whereas the rest of the molecule branches out in a flexible extended conformation (*yellow*, UAS; *gray*, ubiquitin-like domain; *red*, UBA). p47 also binds to p97 in a 3:6 ratio via the UBX site, p47 oligomerizes through SEP domains (*teal*), and it also has a UBA domain in an extended fashion. UN (*purple*) binds two binding sites but in the same conformation of p97. Common features between p97-adaptor complexes may be part of a general mode of p97 adaptor binding, where the adaptor (*light orange*) binds the p97 N-terminal domains (*blue circles*) rotated over the top of the molecule and via multiple sites.

The p97-FAF1 complex has a number of features that are seen in other p97-adaptor complexes, suggesting a common mode of p97 binding to these adaptor proteins (Fig. 6). This common binding mode involves the binding of the adaptor protein to the p97 N-terminal domains above the double-AAA rings and via multiple N-terminal domains to form a p97-adaptor conformation. Their extended and polyvalent nature facilitates their functions in targeting specific components within a large assembly or embedded in membranes or chromatin. A common mode of binding suggests that p97 preferentially binds to a single cofactor at a time, determined by the increased local concentration of the cofactor, to ensure substrates specificity in mediating its diverse array of multiprotein complexes in a highly specific fashion.

## Footnotes

2 The abbreviations used are:

AAA: ATPases associated with various cellular activities
cryo-EM: cryo electron microscopy
ERAD: endoplasmic reticulum-associated degradation
FAF1: Fas-associated factor 1
UBX: ubiquitin-associated domain X
UBA: ubiquitin-associating
MST: microscale thermophoresis
CC: coiled coil
SV-AUC: sedimentation velocity ultra-centrifugation
ITC: isothermal titration calorimetry
UN: ubiquitin fusion degradation 1 (Ufd1)-nuclear protein localization 4(Npl4).
